# Neurocardiac consequences of traumatic brain injury: integrating neuroimmune, autonomic, and cerebrovascular mechanisms

**DOI:** 10.3389/fimmu.2026.1861951

**Published:** 2026-06-03

**Authors:** Claymore T. Gumbo, Mikaela A. Barbour, Zachary M. Weil

**Affiliations:** 1 Department of Neuroscience and Rockefeller Neuroscience Institute, West Virginia University, Morgantown, WV, United States; 2 Department of Pharmaceutical Sciences, School of Pharmacy, West Virginia University, Morgantown, WV, United States

**Keywords:** autonomic dysregulation, brain-heart axis, cardiac injury, cerebrovascular dysfunction, neurocardiac dysfunction, neuroinflammation, traumatic brain injury

## Abstract

Traumatic brain injury (TBI) is increasingly recognized as a systemic disorder with consequences that extend beyond the central nervous system (CNS) to include clinically relevant cardiac dysfunction. Clinical and experimental evidence indicate that TBI is associated with arrhythmias, myocardial injury, autonomic imbalance, and impaired cardiac performance, even in the absence of primary cardiac disease. These observations support the concept of a brain-heart axis through which neural injury influences cardiovascular regulation. Current evidence shows that multiple interacting mechanisms contribute to cardiac abnormalities following TBI. Acute autonomic dysregulation, particularly sympathetic overactivation, is strongly supported as an early driver of cardiovascular instability, while neuroimmune and neuroendocrine responses may modulate the persistence and severity of downstream effects. Emerging evidence further suggests that cerebrovascular dysfunction may act as a modifier of neurocardiac vulnerability, although direct clinical evidence remains limited. At the myocardial level, processes including inflammatory signaling, oxidative stress, and mitochondrial dysfunction are associated with electrophysiologic instability and impaired contractile function. However, much of the mechanistic understanding derives from experimental models, and translation to human populations remains incomplete. Accordingly, cardiac involvement after TBI is best understood as a multifactorial and heterogeneous process rather than the consequence of a single dominant pathway. This review synthesizes current clinical and experimental evidence within an integrated framework, emphasizing the interplay among autonomic, immune, and vascular mechanisms. Improved understanding of these interactions may enhance risk stratification and support the development of targeted strategies to mitigate cardiovascular complications after TBI.

## 1 Introduction

Traumatic brain injury (TBI) is a significant worldwide public health problem and a leading cause of disability and mortality across the lifespan (1). An estimated 69 million people experience a TBI annually worldwide, arising from road traffic accidents, falls, sport-related injuries, violence, and during military exposure (2). In the United States alone, approximately 2.8 million TBI-related emergency department visits, hospitalizations, and deaths occur each year, with incidence expected to rise as the population ages and exposure to injury risk increases (3). Although TBIs are commonly classified as mild, moderate, or severe based on clinical criteria, these categories do not always predict the extent of extracranial or long-term systemic consequences, which frequently involve other organ systems and contribute to chronic disability (4).

Historically, TBI has been conceptualized primarily as a disorder of the central nervous system (CNS), with emphasis placed on cognitive impairment, affective disturbances, and neurodegenerative risk. However, a growing body of evidence suggests that TBI is better understood as a systemic disease, with sustained effects on immune, endocrine, and cardiovascular function. Among these extracranial consequences, cardiac dysfunction has emerged as a clinically significant yet underrecognized component of TBI pathophysiology (4–6).

Over the past two decades, both clinical evidence and experimental studies have suggested a clear association between TBI and cardiovascular dysfunction. Survivors of TBI exhibit increased incidence of arrhythmias, myocardial injury, autonomic dysregulation, and impaired cardiac output (7). Importantly, these effects are not limited to severe injury; even mild TBI has been associated with measurable alterations in cardiac function, particularly in individuals with pre-existing risk factors (8). These observations have expanded the classical framework of TBI to include the brain-heart axis (9), a bidirectional network linking neural injury to cardiovascular dysfunction.

Despite this growing recognition, the true prevalence and clinical significance of post-TBI cardiac dysfunction remain difficult to define. Variability in injury severity, timing of assessment, and diagnostic criteria complicates interpretation across studies. Moreover, cardiac abnormalities are frequently subclinical or “silent,” escaping detection by standard clinical evaluation while nonetheless contributing to morbidity, impaired rehabilitation, and, in severe cases, mortality (10–12). This raises the possibility that cardiac dysfunction represents a pervasive but underappreciated determinant of long-term outcome following TBI.

Multiple, partially overlapping mechanisms have been proposed to account for TBI-induced cardiac dysfunction. Disruption of the central autonomic network (CAN), particularly through sympathetic overactivation, is widely considered a major contributor (13, 14). In parallel, neuroinflammation, neuroendocrine dysregulation, and injury to key autonomic regulatory regions further destabilize cardiovascular control (15, 16). However, these mechanisms are typically considered in isolation, and an integrative framework that explains variability in cardiac outcomes across individuals remains lacking.

Increasing evidence suggests that cerebrovascular integrity may represent an important and underappreciated modifier of neurocardiac interactions following TBI. TBI-induced impairments in cerebral blood flow (CBF), blood-brain barrier (BBB) function, and cerebrovascular reactivity contribute not only to secondary brain injury but also to dysfunction within autonomic control centers (17–19). Microvascular damage can persist for weeks to months after injury, potentially altering the neural circuits that regulate cardiovascular function (20). Regions such as the insular cortex, which are highly sensitive to hypoperfusion and play a central role in autonomic regulation, may serve as key interfaces linking cerebrovascular dysfunction to cardiac instability (21, 22).

An important and largely unresolved question is whether pre-existing cerebrovascular compromise, as occurs in conditions such as hypertension, diabetes, and atherosclerosis, primes the brain-heart axis for exaggerated dysfunction following TBI. Experimental models of chronic cerebral hypoperfusion, including bilateral carotid artery stenosis (BCAS), suggest that reduced vascular reserve limits the brain’s capacity to respond to injury, amplifies neuroinflammatory signaling, and exacerbates downstream physiological dysfunction (23, 24). These findings suggest that impaired cerebrovascular reserve (CVR) may not only worsen neurological outcomes but also potentiate cardiac dysfunction through sustained autonomic imbalance and systemic inflammatory signaling.

Increasing evidence indicates that inflammatory signaling triggered by TBI is not confined to the injured brain but extends systemically through circulating cytokines, immune cell mobilization, and vascular dysfunction. These responses can alter peripheral organ function, creating conditions that sensitize tissues such as the myocardium to secondary injury (25, 26). Clinical and experimental studies have shown that systemic inflammatory mediators released after TBI contribute to multi-organ dysfunction, including cardiovascular abnormalities (27, 28). Viewing post-traumatic cardiac dysfunction through this broader immunological framework provides a useful perspective for understanding how central injury translates into persistent peripheral pathology.

While previous reviews have described cardiovascular complications following TBI, many have focused on individual mechanisms such as autonomic dysregulation or neuroinflammatory signaling in isolation. The present review extends beyond descriptive summaries by proposing an integrated neuroimmune-vascular-autonomic framework that positions cerebrovascular integrity, particularly CVR, as a potential modifier of neurocardiac vulnerability. By linking central autonomic disruption with vascular resilience and systemic immune activation, this manuscript highlights mechanistic intersections that may explain the heterogeneity of cardiac outcomes observed after TBI. This integrative perspective aims to generate testable hypotheses and identify emerging biomarkers and therapeutic targets that are not fully addressed in existing reviews. In particular, the emphasis on CVR as a systems-level modifier provides a conceptual bridge between neural injury, immune activation, and cardiac vulnerability that has not been systematically synthesized in prior literature.

In this review, neuroimmune processes refer to the coordinated activation of central and peripheral immune signaling pathways following TBI, including microglial activation, cytokine release, and systemic immune responses. CVR refers to the capacity of cerebral vessels to maintain adequate perfusion, BBB function, and vascular responsiveness under physiological stress.

## 2 Literature search and selection strategy

This review was conducted as a qualitative synthesis of clinical and experimental literature examining mechanisms linking TBI to cardiovascular dysfunction. Relevant studies were identified through structured searches of major biomedical databases, including PubMed, Scopus, and Web of Science, covering publications from January 2000 through March 2026. Additional articles were identified through manual review of reference lists from key primary studies and review articles. Studies were initially screened based on title and abstract relevance, followed by full-text evaluation for mechanistic and clinical relevance to neurocardiac dysfunction following TBI.

Studies were included if they examined cardiovascular, autonomic, inflammatory, cerebrovascular, or myocardial outcomes following clinical or experimental TBI. Both human and animal studies were considered in order to integrate mechanistic insights with clinically relevant observations. Priority was given to studies providing mechanistic, physiological, molecular, or imaging-based evidence linking CNS injury to cardiac outcomes. Reviews and meta-analyses were used to identify foundational concepts and emerging mechanistic pathways. Study selection was guided by relevance to the central objective of integrating autonomic, neuroimmune, and cerebrovascular mechanisms underlying neurocardiac dysfunction.

Because the objective of this manuscript is to provide a mechanistic and hypothesis-generating framework rather than a quantitative synthesis, this review was conducted as a structured narrative synthesis rather than a formal systematic review. Accordingly, formal meta-analysis and structured evidence grading were not performed, and study selection was guided by conceptual relevance rather than exhaustive inclusion criteria. Emphasis was placed on identifying converging patterns of evidence across clinical and experimental studies supporting integrated neuroimmune, autonomic, and cerebrovascular mechanisms underlying neurocardiac dysfunction following TBI. Where appropriate, conflicting findings and studies reporting heterogeneous or limited associations were intentionally included to better reflect uncertainty and variability within the field. Distinctions were made between observational clinical findings, experimentally derived mechanistic insights, and hypothesis-generating concepts. Accordingly, conclusions should be interpreted within the context of this qualitative, hypothesis-generating narrative framework.

## 3 Current clinical evidence linking TBI and cardiac dysfunction

To ground this conceptual framework, it is essential to examine the clinical evidence demonstrating that TBI is associated with reproducible cardiac dysfunction. Across injury severities, patients exhibit arrhythmias, myocardial injury, and impaired cardiac performance, supporting the clinical relevance of the brain-heart axis. However, these observations also display substantial heterogeneity, suggesting that additional modifiers, such as cerebrovascular integrity and baseline vascular health, likely influence individual susceptibility to neurocardiac complications.

**Figure 1:**
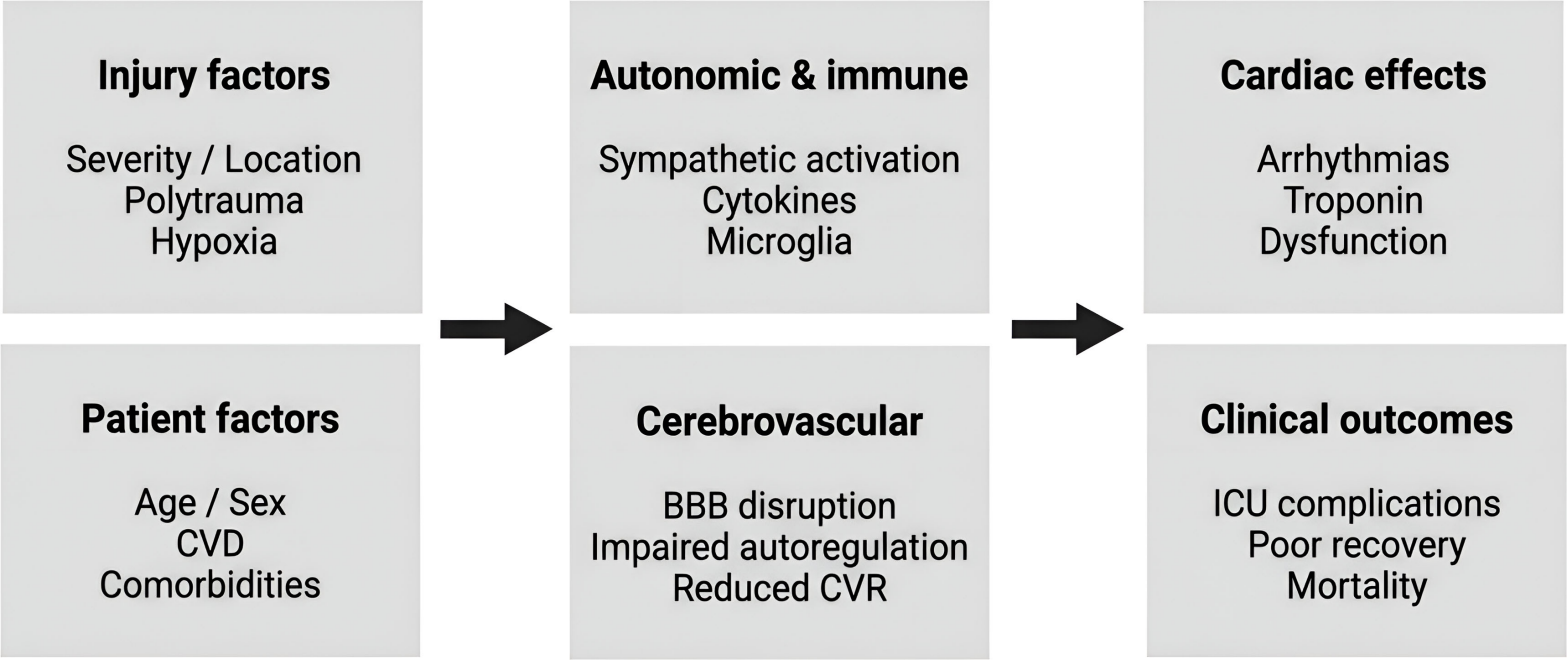
Conceptual framework diagram illustrating sources of heterogeneity influencing neurocardiac dysfunction following TBI. Predisposing factors include injury severity, injury location, polytrauma, hypoxia, age, sex, pre-existing cardiovascular disease, and comorbidities. Mechanistic domains include autonomic and neuroimmune processes such as sympathetic activation, cytokine signaling, and microglial activation, as well as cerebrovascular dysfunction including BBB disruption, impaired autoregulation, and reduced CVR. Downstream cardiac manifestations include arrhythmias, troponin elevation, and myocardial dysfunction, which may contribute to intensive care unit complications, poor recovery, and mortality.

Clinical evidence increasingly shows that TBI is associated with both acute and persistent cardiovascular abnormalities (**Figure 1**). Cardiac sequelae occur in the immediate post-injury period as well as during recovery and include arrhythmias, myocardial injury, and, in severe cases, cardiac arrest (18). Up to one-third of patients with severe TBI develop arrhythmias, most commonly atrial fibrillation, ventricular tachyarrhythmias, and QTc prolongation (29, 30). Importantly, ventricular arrhythmias and cardiac arrest are associated with worse neurological outcomes and increased mortality in neurocritical care populations, while broader autonomic and neurocardiovascular literature supports a potential role for dysregulated sympathetic signaling in these complications (31). These findings are consistent with disruption of autonomic regulation, in which excessive sympathetic activation and catecholamine surges may contribute to cardiac electrophysiology.

Biomarker studies further support the presence of myocardial injury following TBI. Elevated serum troponin levels are frequently observed in patients with moderate to severe injury, even in the absence of underlying coronary artery disease (10, 32). Importantly, observational evidence from critically ill populations, including patients with neurological injury, suggests that troponin elevation is associated with adverse outcomes such as increased mortality, prolonged intensive care unit (ICU) stay, and poorer recovery (33). To clarify the strength, timing, and limitations of available human evidence, we summarize human and translational evidence relevant to neurocardiac dysfunction following TBI in **Table 1**.

**Table 1:** Representative human and translational evidence relevant to neurocardiac dysfunction following TBI.

Study	Evidence type	Population/model	Principal findings	Major limitations/confounders	Relevance to neurocardiac dysfunction
10	Systematic review and meta-analysis	Adult TBI cohorts	Myocardial injury and cardiac dysfunction are frequently reported after TBI and are associated with worse clinical outcomes	Heterogeneity in cardiac definitions, injury severity, and timing of assessment across included studies	Direct evidence supporting the clinical relevance of post-TBI cardiac abnormalities
7	Narrative/translational review	Neurocritical care and TBI literature	Summarized autonomic dysregulation, catecholamine excess, and neurogenic myocardial dysfunction after neurological injury	Primarily mechanistic synthesis rather than prospective cohort data	Contextual evidence supporting autonomic mechanisms linking TBI and cardiac dysfunction
29	Primary clinical cohort	Pediatric severe TBI population undergoing hypothermia therapy	Cardiac arrhythmias frequently occurred after severe TBI	Pediatric population: hypothermia may independently influence arrhythmia risk	Clinical evidence of electrophysiologic instability in severe pediatric TBI populations
8	Population-based cohort study	Patients with prior TBI	TBI was associated with increased long-term risk of heart failure and coronary heart disease	Association-based design with limited mechanistic specificity	Supports potential long-term cardiovascular consequences following TBI
18	Narrative clinical review	Neurocritical care and TBI literature	Summarized myocardial injury, arrhythmias, autonomic dysfunction, and management considerations following TBI	Review-level evidence rather than primary prospective clinical data	Integrative clinical framework for neurocardiac dysfunction following TBI
33	Critical care observational cohort	Mixed critically ill ICU population including neurological injury	Elevated troponin levels were associated with increased mortality risk in critically ill populations	Not specific to isolated TBI populations	Contextual evidence supporting the prognostic significance of myocardial injury biomarkers
30	Observational clinical study	Severe TBI populations	QTc prolongation and arrhythmias were associated with adverse neurologic outcomes	Potential confounding by sedation, ventilation, and catecholamine exposure	Direct evidence linking autonomic instability and electrophysiologic abnormalities after TBI
34	Systematic review and meta-analysis	Acute TBI populations	β-blocker exposure was associated with reduced mortality after TBI	Predominantly observational data with potential treatment-selection bias	Supports the hypothesis-generating therapeutic relevance of sympathetic modulation following TBI

TBI, traumatic brain injury; ICU, intensive care unit; QTc, corrected QT interval.

This table is intended as an illustrative summary of representative evidence rather than a formal, quantitative evidence-grade extraction table. Findings should be interpreted within the context of varying injury severity, study design, and potential confounders.

Studies are categorized according to evidence type, study population, principal findings, major limitations/confounders, and relevance to neurocardiac dysfunction following TBI. The table includes primary clinical cohorts, population-based studies, systematic reviews/meta-analyses, observational critical care studies, and contextual translational literature to provide a qualitative overview of current evidence linking autonomic dysregulation, neuroimmune activation, cerebrovascular dysfunction, and cardiac abnormalities after TBI. Because available evidence remains heterogeneous and largely observational, findings should be interpreted within the context of varying injury severity, intensive care interventions, cardiovascular monitoring strategies, and study design limitations.

Although the available literature shows consistent associations between TBI and cardiac abnormalities, most clinical studies remain observational in design and are subject to important confounding variables, including injury severity, hypoxia, pharmacologic interventions, mechanical ventilation, and systemic inflammatory responses. Consequently, while mechanistic pathways linking brain injury to cardiac dysfunction have been shown in controlled experimental settings, causal relationships in human populations remain incompletely defined. In this review, clinical observations are interpreted as associative evidence, while mechanistic inferences are primarily derived from experimental studies and considered within their appropriate evidentiary context.

This distinction is particularly challenging in clinical settings, where systemic factors such as hypoxia, hypotension, mechanical ventilation, sedative exposure, and exogenous catecholamine administration may independently alter cardiac physiology. As a result, observed cardiac abnormalities likely reflect a combination of primary neurogenic effects and secondary systemic influences, complicating attribution to a single mechanism.

**Figure 2:**
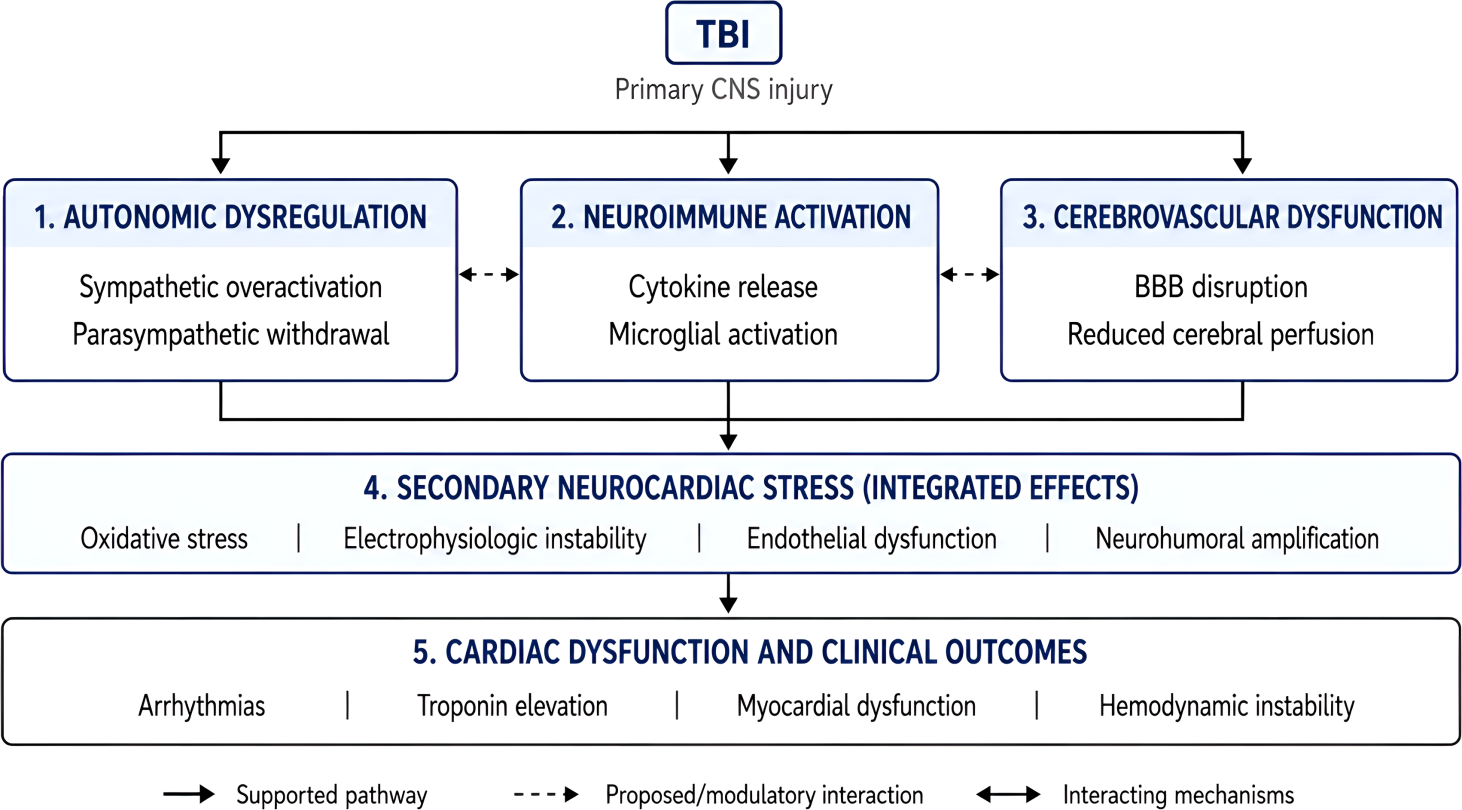
Mechanistic framework diagram illustrating proposed interacting pathways linking TBI to cardiac dysfunction. Acute TBI may trigger autonomic dysregulation, neuroimmune activation, and cerebrovascular dysfunction through interconnected mechanisms involving sympathetic overactivation, cytokine signaling, BBB disruption, impaired autoregulation, and reduced CVR. These pathways may contribute to secondary neurocardiac stress characterized by oxidative stress, electrophysiologic instability, endothelial dysfunction, and neurohumoral amplification, potentially contributing to cardiac dysfunction and adverse clinical outcomes. Solid arrows indicate supported pathways, dashed arrows indicate proposed or modulatory interactions, and bidirectional arrows represent interacting mechanisms.

Taken together, these observations support the view that TBI can be understood as a systemic, multi-organ process in which cardiac dysfunction may contribute meaningfully to patient outcomes. From a clinical perspective, these findings suggest a potential role for routine cardiac monitoring, including electrocardiogram (ECG) and biomarker assessment, into the management of acute TBI, with the goal of improving risk stratification and mitigating secondary cardiac injury. At the same time, the variability in cardiac manifestations across patients underscores the need to better understand the upstream mechanisms that govern susceptibility to neurocardiac dysfunction. While these clinical observations support the presence of neurocardiac dysfunction, they do not fully explain the underlying biological mechanisms. These mechanistic pathways are explored in **Figure 2**.

## 4 Experimental insights from preclinical models

Preclinical models have been instrumental in defining the mechanistic links between TBI and cardiac dysfunction, providing experimental control and tissue-level resolution that are not readily achievable in human studies. Across diverse injury paradigms, animal models consistently show that brain injury alone is associated with measurable cardiac abnormalities, even in the absence of pre-existing cardiovascular disease or direct myocardial trauma (26, 35). These findings support the interpretation that neurocardiac dysfunction can arise in response to TBI under controlled experimental settings, rather than solely as a secondary effect of comorbidity or critical illness.

Rodent models of controlled cortical impact, fluid percussion injury, and blast exposure reveal early and persistent alterations in cardiac structure and function following TBI. These include reductions in left-ventricular ejection fraction, impaired fractional shortening, and reduced cardiac output, often detectable within days of injury and persisting for weeks to months thereafter (21, 26). Importantly, these functional impairments are accompanied by cardiomyocyte apoptosis, inflammatory infiltration, and fibrotic remodeling, evidence consistent with a mechanistic contribution between central injury and peripheral myocardial pathology.

Experimental studies further distinguish between functional and structural cardiac impairments, suggesting that neurogenic mechanisms may precede, and potentially drive, long-term remodeling. In several models, systolic dysfunction and electrophysiologic instability emerge before overt histological damage, indicating that altered autonomic drive and neurohumoral signaling may acutely disrupt myocardial performance (7, 36). Over time, persistent immune activation and oxidative stress promote cardiomyocyte loss and extracellular matrix deposition, transitioning the phenotype from reversible dysfunction to more durable structural injury (37). This temporal sequence supports a model in which early autonomic and neurohumoral disturbances act as important early contributors, while inflammation and remodeling represent downstream amplifiers of cardiac pathology.

Importantly, interventional studies provide evidence implicating specific brain-derived signaling pathways in cardiac injury. Attenuation of sympathetic signaling via β-blockade reduces myocardial oxidative stress and preserves cardiac function after TBI, supporting a mechanistic role for catecholaminergic overactivation in myocardial injury (36, 38). Similarly, splenectomy markedly reduces myocardial inflammation and fibrosis, demonstrating a critical role for systemic immune signaling in mediating post-TBI cardiac pathology (26). Collectively, these findings support important roles for both autonomic and immune pathways in the development and persistence of cardiac dysfunction following brain injury.

However, several limitations of preclinical models should be considered. Most studies are conducted in young, otherwise healthy animals and may not capture the influence of aging, comorbid vascular disease, or preexisting cerebrovascular dysfunction, all of which are highly relevant in clinical populations. In addition, variability in injury models and outcome measures complicates direct comparison across studies. These limitations highlight the need for experimental approaches that incorporate vascular and systemic modifiers of injury, particularly those affecting cerebrovascular integrity and reserve. Accordingly, mechanistic conclusions derived from experimental models should be interpreted as providing causal insight under controlled conditions, while translation to human neurocardiac outcomes remains inferential and requires clinical validation.

## 5 Neuroanatomical basis of brain-heart interaction

The neural control of cardiovascular function is mediated by a distributed and hierarchically organized network of brain regions collectively referred to as the CAN. This network integrates viscerosensory input, emotional and cognitive context, and systemic physiological signals to dynamically regulate heart rate, myocardial contractility, and vascular tone (39, 40). While this architecture enables flexible cardiovascular control under normal conditions, it also creates multiple points of vulnerability through which brain injury can disrupt cardiac function. The CAN integrates peripheral sensory input with signals from cortical and subcortical regions to regulate cardiovascular function. Key structures, including the nucleus of the solitary tract, hypothalamus, and limbic regions, coordinate autonomic responses to physiological and environmental stressors (41). These networks modulate sympathetic and parasympathetic outflow through brainstem and spinal pathways, allowing rapid adjustment of cardiovascular function (42).

Within this network, specific regions appear particularly critical for neurocardiac regulation and disproportionately relevant to TBI. Among these, the insular cortex has emerged as a central hub linking cortical processing to autonomic control. The insula integrates viscerosensory information with emotional and cognitive state and exerts modulatory control over sympathetic and parasympathetic output (43, 44). Lesion and stimulation studies in humans suggest that disruption of insular function can shift sympathovagal balance and promote arrhythmogenesis, highlighting its role as a key node in cardiac regulation.

Importantly, the vulnerability of the CAN is not determined solely by its anatomical organization, but also by the cerebrovascular environment in which it operates. Regions such as the insular cortex and brainstem autonomic nuclei are highly sensitive to changes in perfusion and metabolic supply. Even modest reductions in CBF or CVR may impair the function of these autonomic centers, altering their ability to regulate cardiovascular output (22). This raises the possibility that cerebrovascular dysfunction, whether induced by TBI or present prior to injury, may selectively destabilize key nodes within the CAN, thereby amplifying downstream cardiac consequences.

## 6 Peripheral pathology: cardiac structural and functional outcomes

Clinical and experimental evidence consistently show that TBI is associated with a distinct pattern of peripheral cardiac abnormalities, suggesting that brain injury may contribute to myocardial dysfunction in addition to secondary systemic effects. Patients and animal models exhibit myocardial injury, ventricular dysfunction, arrhythmias, and characteristic regional wall motion abnormalities, indicating that TBI may contribute to a distinct pattern of myocardial dysfunction beyond secondary systemic effects (10, 45). Importantly, these peripheral changes reflect the downstream consequences of sustained neuroimmune activation, which interacts with autonomic disruption and neurohumoral signaling to drive myocardial injury.

### 6.1 Structural remodeling: cardiomyocyte injury, apoptosis, and fibrosis

Structural cardiac remodeling following TBI is characterized by early cardiomyocyte injury and progressive fibrotic transformation. Available evidence from experimental models supports the interpretation that TBI induces cardiomyocyte apoptosis within days of injury, accompanied by oxidative stress, inflammatory signaling, and cellular hypertrophy (26). These early changes are followed by ventricular dilation and extracellular matrix remodeling, with increased collagen deposition driven by persistent immune activation and fibroblast-myofibroblast transition.

Mechanistically, these processes are consistent with a model in which neurogenic and systemic signals initiate myocardial injury, while immune-mediated pathways sustain and amplify structural remodeling. Circulating inflammatory mediators and infiltrating immune cells, including macrophages, neutrophils, and mast cells, promote fibroblast activation through profibrotic signaling pathways such as transforming growth factor-β (TGF-β), leading to progressive myocardial stiffening and impaired compliance (46) (47). While these features resemble fibrosis observed in other forms of sterile cardiac injury, their emergence in the absence of primary cardiac insult underscores the capacity of TBI to initiate maladaptive remodeling through brain-derived mechanisms.

### 6.2 Electrophysiological and contractile dysfunction

Functional cardiac impairment following TBI reflects both acute neurogenic disruption and evolving structural changes. Clinically, patients exhibit QTc prolongation, arrhythmias, and regional wall motion abnormalities, with conduction disturbances occurring several-fold more frequently than in non-TBI populations. Preclinical models parallel these findings, demonstrating reduced fractional shortening, decreased left ventricular ejection fraction, and impaired systolic function independent of preexisting cardiac disease (18).

Notably, patterns of myocardial dysfunction following TBI often differ from classic ischemic injury. In neurologically injured populations, including brain death, a substantial proportion of patients exhibit systolic dysfunction with segmental patterns that spare the apex, a finding consistent with neurogenic stunned myocardium rather than coronary artery occlusion. This distinction is mechanistically important: neurogenic cardiac injury arises from catecholamine-mediated toxicity, calcium overload, and regional differences in adrenergic receptor density, rather than from ischemia due to obstructive coronary disease. These processes may promote electrophysiologic instability and increase susceptibility to ventricular arrhythmias, suggesting a link between central autonomic dysregulation and cardiac dysfunction (48).

### 6.3 Immune-mediated mechanisms in myocardial injury

Immune activation represents an important mechanistic link between central injury and sustained peripheral cardiac pathology. Experimental studies suggest that TBI triggers recruitment of circulating immune cells, including splenic-derived leukocytes, into the myocardium, where they amplify oxidative stress, inflammatory signaling, and apoptotic pathways within cardiomyocytes. Interventions such as splenectomy markedly attenuate myocardial inflammation, fibrosis, and cell death, providing evidence that systemic immune responses contribute to TBI-induced cardiac injury (26).

Beyond cellular infiltration, circulating cytokines and damage-associated molecular patterns propagate inflammatory signaling to the heart. Mediators such as IL-1β, TNF-α, complement factors, and extracellular histones promote fibroblast activation, extracellular matrix remodeling, and electrophysiologic instability, recapitulating mechanisms observed in other forms of inflammatory cardiomyopathy (37, 47). In this context, the immune response likely contributes not only as a downstream consequence of TBI, but also as a sustained amplifier of cardiac pathology.

These structural, functional, and immune-mediated changes collectively support the presence of a peripheral cardiac phenotype following TBI. This phenotype arises from the interaction of early autonomic and neurohumoral disruption with sustained inflammatory and oxidative mechanisms, resulting in progressive myocardial injury and dysfunction. As such, peripheral cardiac pathology represents the downstream arm of the brain-heart axis and provides a mechanistic link between central injury and systemic clinical outcomes. These findings underscore the importance of incorporating cardiac monitoring and cardioprotective strategies into TBI management, while also highlighting the need to identify upstream factors, such as cerebrovascular dysfunction, that may modulate the severity of cardiac involvement.

## 7 Central mechanisms contributing to cardiac dysfunction

TBI can initiate a coordinated neuroimmune response that may disrupt central autonomic and neuroendocrine networks essential for cardiovascular homeostasis (25, 49). These processes represent interacting and partially overlapping contributors to neurocardiac dysfunction following TBI, rather than a strictly hierarchical cascade (**Figure 2**). While acute autonomic dysregulation, particularly sympathetic overactivation, is supported as an initiating mechanism, neuroimmune and neuroendocrine pathways likely act as modulators that shape the magnitude and persistence of downstream cardiac effects.

### 7.1 Sympathetic overactivation and autonomic imbalance (sympathetic storm)

One of the earliest and most extensively characterized central responses to TBI is acute sympathetic overactivation. TBI triggers rapid and disproportionate activation of the sympathetic nervous system through hypothalamic-brainstem circuits and descending spinal pathways, driving surges of norepinephrine and epinephrine from both cardiac sympathetic terminals and the adrenal medulla. This catecholaminergic storm may produce tachyarrhythmias, QTc prolongation, and transient ventricular dysfunction (neurogenic stunned myocardium), sometimes extending to Takotsubo-like phenotypes (50, 51).

Mechanistically, excessive β-adrenergic stimulation increases myocardial oxygen demand, calcium loading, and cyclic AMP signaling, predisposing to contraction band necrosis, repolarization instability, and malignant ventricular arrhythmias. These changes have been consistently described following neurological injury, including TBI, and are consistent with features of an early neurocardiac phenotype (7, 52).

Autonomic imbalance frequently persists beyond the acute phase, characterized by reduced parasympathetic tone and sustained sympathetic predominance. This is reflected in decreased heart-rate variability and impaired baroreflex sensitivity weeks to months after injury, with greater severity associated with more pronounced long-term dysfunction (53, 54). Within the CAN, the insula and hypothalamus are key cortical and subcortical hubs regulating cardiovascular output. The insular cortex exhibits hemispheric and topographic specialization for heart rate and rhythm control, and human lesion and stimulation studies suggest that disruption of insular circuits promotes sympathovagal imbalance and arrhythmogenesis (55, 56).

Brainstem autonomic nuclei, including the nucleus tractus solitarius, dorsal motor nucleus of the vagus, nucleus ambiguus, and rostral ventrolateral medulla, integrate baroreceptor inputs and generate sympathetic and parasympathetic output. Injury or neuroinflammatory activation within these regions can blunt baroreflex sensitivity, reduce vagal efferent activity, and reinforce sustained sympathetic drive. Together, dysfunction across the insula-hypothalamus-brainstem axis may contribute to persistent dysautonomia after TBI (57–59). Importantly, autonomic dysregulation and neuroinflammatory signaling likely interact bidirectionally following TBI, with sympathetic overactivation promoting immune activation while inflammatory processes within autonomic centers further destabilize cardiovascular regulation.

### 7.2 Neuroinflammation in central autonomic centers

Neuroinflammation is a defining feature of TBI and may act as a key contributor to sustained autonomic dysregulation. Converging evidence suggests that TBI induces activation of microglia and astrocytes within central autonomic regions, including the insula, hypothalamus, and brainstem, altering synaptic transmission, neuromodulator release, and circuit excitability. Neuronal and glial injury following TBI results in the release of damage-associated molecular patterns (DAMPs), including high-mobility group box 1 (HMGB1), extracellular ATP, heat shock proteins, and mitochondrial DNA. These endogenous danger signals activate pattern-recognition receptors such as Toll-like receptors (TLRs) and purinergic receptors on resident microglia and astrocytes, initiating innate immune signaling cascades that amplify local cytokine production and propagate inflammatory signaling beyond the initial injury site (60–62).

Cytokine signaling (e.g., IL-1β, TNF-α), prostanoids, and TGF-β pathways collectively reshape autonomic output, promoting persistent sympathetic activation and impaired vagal regulation (63). Activation of inflammasome complexes, particularly the NLRP3 inflammasome, represents an important downstream component of TBI-associated neuroinflammation. Inflammasome activation promotes cleavage of pro-interleukin-1β (IL-1β) and interleukin-18 (IL-18), amplifying inflammatory signaling and contributing to BBB disruption and sustained autonomic instability (64, 65).

Notably, neuroinflammation may interact bidirectionally with vascular and metabolic stress. In experimental models, TBI is associated with insular gliosis, and pre-existing cerebral hypoperfusion enhances glial activation in the insula even in the absence of overt cardiac dysfunction, providing a mechanistic link among cerebrovascular stress, neuroinflammation, and dysautonomia (21). These findings position neuroinflammation as a sustained amplifier of autonomic dysfunction rather than an isolated process. Consistent with this, clinical and translational studies identify neuroinflammation as a key modifier of paroxysmal sympathetic hyperactivity and cardiovascular instability following TBI (66).

Although neuroinflammatory processes are consistently observed following TBI and may contribute to sustained autonomic and myocardial dysfunction, current evidence does not support their interpretation as a singular dominant driver, but rather as part of a broader network of interacting mechanisms.

### 7.3 Hypothalamic-pituitary-adrenal axis dysregulation and stress hormone signaling

TBI also disrupts the hypothalamic-pituitary-adrenal (HPA) axis through injury to hypothalamic neurons, pituitary structures, and limbic feedback pathways. Clinical and experimental studies show post-TBI hypopituitarism and instability in glucocorticoid signaling, including both hyperactivation and secondary adrenal insufficiency, with severity scaling to injury burden (67).

Maladaptive HPA activity further amplifies autonomic imbalance, alters myocardial substrate utilization, and promotes systemic inflammation, increasing susceptibility to arrhythmia and impaired cardiac reserve. Importantly, HPA dysregulation interacts with neuroinflammatory signaling to create a feed-forward loop that sustains both autonomic and inflammatory dysfunction over time (68).

### 7.4 Oxidative stress, mitochondrial injury, and electrophysiologic instability

Central autonomic and inflammatory signals converge on the myocardium, where they induce oxidative stress and mitochondrial dysfunction. Within the myocardium, inflammatory signaling is accompanied by recruitment of immune cells, including macrophages and neutrophils, which contribute to tissue injury through production of reactive oxygen species (ROS), matrix metalloproteinases, and pro-inflammatory cytokines. Macrophage polarization states, including pro-inflammatory (M1-like) and reparative (M2-like) phenotypes, may influence the balance between myocardial injury and repair following neurogenic cardiac stress. In experimental models, myocardial ROS increase within 48 hours after TBI, and β-blockade attenuates this response, linking sympathetic overactivation to oxidative injury (36).

Oxidative stress reduces nitric oxide bioavailability, disrupts excitation-contraction coupling, and promotes calcium handling abnormalities, increasing susceptibility to arrhythmias and contractile dysfunction. These processes represent key downstream effectors of neurogenic cardiac injury and are consistent with mechanisms described in broader inflammatory and stress-induced cardiomyopathy literature (69, 70).

### 7.5 Blood-brain barrier breakdown and systemic propagation of injury signals

TBI-associated BBB disruption permits infiltration of circulating immune cells, including neutrophils, monocytes, and T lymphocytes, into injured brain regions. In parallel, mobilization of immune cells from peripheral reservoirs such as the spleen and bone marrow contributes to systemic inflammatory amplification following injury. These infiltrating leukocytes may produce ROS, proteases, and pro-inflammatory cytokines that exacerbate endothelial dysfunction and destabilize autonomic circuits (71).

Spreading depolarizations and microvascular injury further exacerbate BBB dysfunction, sustaining neurovascular inflammation and propagating maladaptive signaling across central and peripheral systems (72, 73). Complement activation represents an additional mechanism contributing to vascular and tissue injury following TBI. Activation of complement components such as C3a and C5a promotes leukocyte recruitment, endothelial activation, and amplification of inflammatory signaling across neurovascular and myocardial compartments (72). In addition, BBB disruption alters the metabolic and hemodynamic environment of autonomic centers, impairing their ability to regulate cardiovascular responses to physiological stress (74).

### 7.6 Cerebrovascular integrity as a modulator of central autonomic output

Cerebrovascular function critically shapes the vulnerability of autonomic networks to TBI. Regions such as the insular cortex and brainstem autonomic nuclei are highly sensitive to changes in perfusion and metabolic supply. Animal studies suggest that chronic hypoperfusion induces insular gliosis and reduces cerebrovascular reactivity, while combined hypoperfusion and TBI amplify neuroinflammatory signaling within autonomic circuits (75). Clinically, impaired cerebral autoregulation is closely associated with autonomic instability after TBI, suggesting that reduced vascular reserve constrains the brain’s ability to buffer physiological stress (76). These observations support a model in which cerebrovascular dysfunction interacts with neuroimmune signaling pathways to act as a modifier of neurocardiac outcomes.

### 7.7 An integrative brain-heart axis model

The following integrative framework synthesizes evidence across clinical observations, experimental models, and mechanistic inference; however, the strength of evidence varies across these domains and should be interpreted accordingly. These mechanisms can be integrated into a systems-level model of neurocardiac dysfunction following TBI, in which autonomic, neuroimmune, and cerebrovascular pathways interact dynamically rather than operating within a fixed hierarchy (**Figure 2**). Acute sympathetic overactivation may represent an important early initiating contributor, producing immediate cardiac stress and electrophysiologic instability. Neuroinflammation and HPA axis dysregulation sustain and amplify autonomic imbalance through interconnected feed-forward loops. Downstream, oxidative stress and mitochondrial injury drive myocardial dysfunction, while BBB disruption facilitates persistent neurovascular and systemic signaling.

This framework generates several testable predictions. First, early markers of autonomic imbalance should precede measurable myocardial dysfunction in the acute phase. Second, individuals with impaired CVR would be expected to exhibit amplified autonomic instability and increased cardiac vulnerability. Third, longitudinal integration of autonomic, vascular, and cardiac metrics should reveal temporally ordered relationships linking central injury to peripheral outcomes. These predictions provide a basis for future experimental and clinical validation. For example, a prospective clinical study in well characterized, isolated TBI cohorts could longitudinally assess CVR, autonomic function, and cardiac outcomes following TBI. Patients would undergo early assessment of CVR using transcranial Doppler or arterial spin labeling MRI with vasodilatory challenge, alongside continuous ECG monitoring and serial measurement of cardiac biomarkers (e.g., troponin). Autonomic function could be quantified using heart rate variability and baroreflex sensitivity. If the proposed model is correct, impaired CVR would predict greater autonomic instability and increased incidence of myocardial injury or arrhythmias over time, with temporal analyses demonstrating that autonomic disruption precedes measurable cardiac dysfunction. Although such studies are logistically challenging, this approach would allow direct testing of CVR as a modifier of neurocardiac vulnerability in human populations.

Within this framework, cerebrovascular dysfunction emerges as a potential modifier of susceptibility, modulating the extent to which autonomic circuits are disrupted. This framework may help explain why relatively mild injuries can produce significant cardiac effects in vulnerable individuals and why neurogenic cardiac phenotypes often emerge in the absence of obstructive coronary disease (18, 77). Interventions that attenuate sympathetic overactivation (e.g., β-blockade), reduce neuroinflammation, stabilize the BBB, or improve cerebrovascular reactivity may mitigate cardiac dysfunction following TBI. Early translational data are consistent with this concept, suggesting that aspects of neurogenic cardiac dysfunction may be reversible when these pathways are targeted (7, 18).

## 8 Role of cerebrovascular health and cerebrovascular reserve

Beyond neuronal injury, TBI may induce inflammatory and vascular disturbances that impair cerebrovascular function. CVR is typically assessed using imaging modalities such as transcranial Doppler ultrasound, functional MRI, or arterial spin labeling combined with vasodilatory stimuli, allowing quantification of vascular responsiveness to physiological stress (62). Broader neurovascular and autonomic literature suggests that autonomic stability depends on activity-dependent regulation of CBF and intact CVR, the capacity of cerebral vessels to augment perfusion in response to metabolic or pharmacologic demand (78). Disruption of this reserve may therefore sensitize CAN to physiological stress, amplifying downstream cardiovascular dysregulation after TBI. Direct clinical evidence linking impaired CVR to cardiac outcomes following TBI remains limited, and prospective studies integrating vascular and cardiac endpoints are needed to clarify causal relationships. Accordingly, the role of CVR in mediating neurocardiac dysfunction should be considered a hypothesis-generating framework rather than an established determinant, pending validation in prospective clinical studies. While cerebrovascular mechanisms were introduced as modulators of autonomic function in the preceding section, this section focuses specifically on CVR as an independent and potentially measurable determinant of neurocardiac vulnerability.

Both clinical and experimental data indicate that TBI may produce long-lasting disturbances in cerebral perfusion. Neuroimaging studies in blast-exposed veterans show chronically reduced global and regional CBF persisting for years after injury (79). Similarly, murine models of repetitive mild TBI show sustained reductions in CBF lasting months, particularly within watershed regions vulnerable to hypoperfusion (80). These deficits disproportionately affect border-zone regions containing autonomic control centers, including the insular cortex and brainstem, rendering these circuits metabolically vulnerable even in the absence of overt neuronal loss.

Impaired CVR may reduce the physiological resilience of autonomic control regions, potentially amplifying sympathetic activation and increasing susceptibility to cardiovascular instability. In non-TBI populations, atrial fibrillation is associated with impaired CBF and reduced CVR, highlighting a bidirectional relationship between cerebral vascular dysfunction and cardiac pathology (81). These observations suggest that cerebrovascular health may influence cardiac outcomes independently of injury severity. A temporally structured model is therefore proposed in which early vascular impairment following TBI alters autonomic stability, which in turn contributes to downstream myocardial dysfunction during acute and subacute recovery phases.

Experimental evidence from our laboratory supports the hypothesis that CVR may function as an important modifier of brain-heart interactions after TBI. Using bilateral carotid artery stenosis (BCAS) to model chronic cerebral hypoperfusion, we showed that BCAS and TBI independently reduce CBF by approximately 10%, whereas the combination of both insults may produce a larger perfusion deficit accompanied by exacerbated tissue damage and behavioral impairment (82). These findings indicate that pre-existing cerebrovascular compromise amplifies the systemic consequences of TBI and may underlie inter-individual variability in neurocardiac outcomes. While these findings support a mechanistic link between cerebrovascular compromise and injury severity, direct translation to human neurocardiac outcomes remains an important area for future investigation.

At the vascular level, combined BCAS and TBI impair CVR and endothelial integrity. Animals exposed to both insults exhibit a markedly blunted hyperemic response to acetazolamide, consistent with reduced CVR (21, 82). Transcriptomic analyses further reveal endothelial dysregulation, including downregulation of angiogenesis-related pathways and increased extravasation of serum proteins such as IgG and fibrin(ogen), indicative of barrier dysfunction and a propensity for microvascular obstruction (82, 83). Notably, transcriptional changes such as neuronal upregulation of myocardin (MYOCD) suggest engagement of vascular-responsive gene programs in the central response to combined injury.

The CVR framework proposed here generates several testable predictions. First, patients with impaired CVR following TBI would be expected to show greater autonomic instability, as measured by heart rate variability or sympathetic biomarkers. Second, reduced CVR may correlate with increased incidence of myocardial injury, arrhythmias, or contractile dysfunction in both acute and subacute phases of recovery. Third, longitudinal studies integrating cerebrovascular imaging with cardiac monitoring may reveal temporal relationships between vascular impairment and cardiac abnormalities. Testing these predictions will be essential for determining whether CVR functions as a causal mediator or a physiological marker of neurocardiac vulnerability. At present, CVR is best conceptualized as a candidate modifier of neurocardiac vulnerability rather than a confirmed causal mediator.

## 9 Sex differences and biological variability

Evidence regarding sex-specific cardiac outcomes following TBI remains limited; therefore, this section is presented as an exploratory synthesis rather than a definitive conclusion. Although sex differences influence many neurological and cardiovascular conditions, current evidence suggests a more complex, and at times modest, pattern in post-TBI inflammatory and physiological responses.

Experimental studies examining early neuroinflammatory dynamics indicate that males and females often display more similarities than differences following controlled cortical impact. For example, microglial activation, cytokine expression, and immune cell recruitment appear largely comparable between sexes, with only modest divergence in BBB permeability at specific time points (84). These findings challenge the assumption that sex exerts a consistent influence on early TBI-driven immune responses.

At the same time, broader literature suggests that sex may shape TBI outcomes through multiple interacting pathways, including hormonal signaling, inflammatory and oxidative processes, mitochondrial resilience, and behavioral or environmental factors. Some studies propose that females exhibit greater neuroprotective capacity under certain conditions, while others emphasize differences in injury exposure, recovery trajectories, and long-term cognitive outcomes (66, 85). Importantly, these effects are highly context-dependent and do not converge on a single unifying mechanism.

Crucially, most studies of sex differences in TBI focus on neural or systemic endpoints rather than direct cardiac phenotyping. As a result, the extent to which sex influences post-TBI cardiac dysfunction remains largely unresolved. Consistent with this, our previous findings suggest comparable myocardial inflammatory signaling and functional impairment in male and female subjects following TBI (21). These observations suggest that dominant drivers of neurocardiac injury, including sympathetic overactivation, neuroinflammation, and systemic immune signaling, may overshadow more subtle sex-linked biological differences.

Nevertheless, evidence from related contexts indicates that sex can influence key upstream processes relevant to the brain-heart axis. For example, male mice have been reported to exhibit greater peripheral immune cell infiltration and more pronounced microglial activation following cortical impact, along with worse early behavioral outcomes (86). Such differences in inflammatory tone or vascular response could, under specific conditions, translate into sex-dependent cardiac effects, although this possibility remains largely unexplored.

In aggregate, current evidence suggests that the influence of sex on TBI-associated cardiac dysfunction is likely conditional rather than universal. Sex may modulate specific components of the neurocardiac cascade, particularly inflammatory and vascular responses, without fundamentally altering the core mechanisms of autonomic dysregulation and myocardial injury. Resolving these effects will require deliberately designed, sex-inclusive studies that integrate cardiac phenotyping with hormonal, immune, and cerebrovascular measurements. Such approaches will be essential to distinguish true biological differences from those obscured by experimental design or limited endpoints.

## 10 Therapeutic perspectives

Despite increasing recognition of neurocardiac dysfunction as a clinically relevant consequence of TBI, therapeutic strategies specifically targeting cardiac outcomes in this population remain limited. Current management primarily focuses on neurological stabilization, while cardiovascular abnormalities are often addressed as secondary complications. As mechanistic understanding of brain-heart interactions advances, therapeutic approaches can be conceptually organized according to the temporal phases of injury and recovery, including acute stabilization, subacute recovery, and long-term rehabilitation (66).

At present, supportive cardiovascular monitoring, hemodynamic stabilization, and optimization of cerebral perfusion remain the primary evidence-supported clinical approaches for managing neurocardiac dysfunction following TBI. In contrast, most mechanism-targeted interventions discussed below remain experimental, observational, or hypothesis-generating, with limited prospective clinical validation in TBI populations.

### 10.1 Acute phase management: modulating sympathetic overactivation

Interventions targeting sympathetic overactivation represent one of the most clinically accessible strategies during the acute phase of TBI. β-adrenergic blockade has been associated with improved survival and reduced catecholamine-mediated injury in observational studies of severe brain injury. These agents may attenuate myocardial stress, electrophysiologic instability, and contraction band necrosis associated with sympathetic surges (34, 87). However, most available data are observational and subject to confounding, and prospective studies are required to determine the optimal timing, dosing, and patient selection criteria for β-blocker therapy in the context of neurocardiac dysfunction. Importantly, excessive β-adrenergic blockade may worsen hypotension or reduce cerebral perfusion pressure in vulnerable patients, emphasizing the need for careful hemodynamic monitoring, individualized dosing strategies, and prospective clinical evaluation.

### 10.2 Subacute recovery: targeting inflammation and vascular stability

Targeting neuroinflammation represents a complementary strategy aimed at interrupting sustained amplification of autonomic and systemic dysfunction during the subacute phase of recovery. Anti-inflammatory interventions targeting central and peripheral immune responses have shown potential benefit in experimental models of neurological injury (88). These approaches may influence autonomic regulation and reduce myocardial stress; however, translation to clinical practice remains limited by uncertainties regarding timing, specificity, and safety.

Strategies aimed at preserving or restoring cerebrovascular function may represent an additional therapeutic direction. Maintenance of adequate cerebral perfusion and CVR could support the stability of autonomic control regions such as the insula and brainstem (21, 82). Although these strategies remain largely experimental, they align conceptually with models proposing that vascular integrity modulates susceptibility to neurocardiac dysfunction. Further studies integrating cerebrovascular and cardiac endpoints will be necessary to determine whether targeting vascular reserve improves clinical outcomes.

### 10.3 Long-term recovery and prevention: lifestyle and systems-level interventions

Lifestyle-based interventions, including structured physical activity, may provide a systems-level strategy for improving long-term neurocardiac outcomes. Exercise training has been associated with improvements in autonomic balance, vascular function, and myocardial resilience in cardiovascular and neurological populations (89–91). However, evidence specific to TBI remains limited, and variability in injury severity and rehabilitation protocols complicates interpretation. Nevertheless, such interventions offer a potentially practical means of targeting multiple components of the brain-heart axis during recovery.

Despite growing mechanistic insight, most targeted interventions remain supported primarily by preclinical or observational evidence, and prospective clinical trials specifically evaluating neurocardiac outcomes after TBI remain limited. Future clinical studies incorporating standardized cardiac phenotyping, autonomic assessment, and cerebrovascular measurements will be necessary to determine whether mechanism-based interventions translate into meaningful improvements in cardiovascular outcomes following TBI.

## 11 Knowledge gaps and future directions

Despite increasing recognition that TBI is a systemic disorder with consequences extending beyond the CNS, a unifying framework explaining variability in neurocardiac outcomes remains lacking. Although autonomic dysregulation, neuroinflammation, and neuroendocrine disruption are consistently implicated within the brain-heart axis, these processes are often studied in isolation, and the role of cerebrovascular integrity and reserve in shaping vulnerability of autonomic circuits and downstream cardiac dysfunction remains insufficiently defined.

A key priority for future research is to determine whether CVR functions as a central determinant of susceptibility to neurocardiac dysfunction, which will require integrative, longitudinal approaches combining measures of cerebrovascular function with standardized assessments of autonomic balance and cardiac performance. Clarifying the temporal relationships among cerebrovascular dysfunction, autonomic imbalance, and myocardial injury will be essential to distinguish major contributors from downstream amplifiers of pathology.

In addition, the influence of baseline vascular health, including aging and cardiometabolic disease, remains underexplored and should be incorporated into both clinical and experimental studies to improve translational relevance. The development of standardized frameworks integrating autonomic, cerebrovascular, and cardiac metrics will be critical for advancing mechanistic understanding, improving risk stratification, and enabling targeted, mechanism-based interventions.

### 11.1 Emerging hypothesis: cardiomyocyte nuclear stress signatures

In addition to proposed mechanisms of neurocardiac dysfunction, emerging areas of investigation may provide novel insight into early myocardial stress responses following TBI. Although cardiac research has traditionally relied on functional and structural measures to characterize myocardial injury, emerging evidence from other cardiovascular conditions suggests that cardiomyocyte nuclear architecture may serve as a sensitive indicator of early cellular stress. Features such as nucleolar structure, chromatin organization, nuclear volume, and DNA damage have been identified as markers of cellular dysfunction in models of cardiac remodeling (92, 93). The relevance of nuclear stress signaling and cardiomyocyte nuclear remodeling to neurocardiac dysfunction following TBI remains largely unexplored.

Nuclear alterations may represent a downstream readout of integrated neurocardiac stress. Experimental evidence from non-TBI cardiac models suggests that catecholamine excess, oxidative stress, and inflammatory signaling can induce nucleolar disruption, chromatin remodeling, and DNA damage responses within cardiomyocytes (94, 95). Given that similar stressors are present following TBI, it is plausible that nuclear-level changes may occur as part of the myocardial response to neurogenic injury.

At present, direct evidence linking cardiomyocyte nuclear remodeling to TBI-induced cardiac dysfunction remains limited. Therefore, nuclear morphology should be considered a hypothesis-generating domain rather than an established mechanism. Future studies incorporating nuclear-level analyses into experimental models of TBI could help define early biomarkers of cardiac vulnerability and refine mechanistic understanding of brain-heart interactions, as summarized in candidate form in **Table 2**.

**Table 2:** Candidate nuclear features proposed as potential indicators of cardiomyocyte stress based on evidence from non-TBI cardiac models.

Nuclear feature	Biological role	Evidence from non-TBI cardiac models	Potential relevance to TBI	Current evidence in TBI
Nucleolar disruption	Regulates ribosomal biogenesis and protein synthesis	Observed in models of myocardial injury and stress cardiomyopathy; associated with impaired protein synthesis and cellular dysfunction	May reflect early myocardial stress triggered by catecholamine excess or oxidative injury	Not yet studied in TBI-specific cardiac models
Chromatin remodeling	Controls gene accessibility and transcriptional regulation	Linked to cardiac hypertrophy and remodeling through histone modification and chromatin condensation	May reflect transcriptional adaptation to neurogenic cardiac stress	Limited mechanistic and observational evidence
DNA damage markers (γH2AX)	Signals DNA double-strand breaks and genomic instability	Increased in hypertrophic and ischemic cardiac injury models	May indicate oxidative or inflammatory injury in cardiomyocytes after TBI	No direct TBI-specific cardiac data available
Altered nuclear morphology	Reflects structural nuclear stress	Observed in pressure overload and myocardial injury models	May correlate with mechanical and metabolic stress after neurogenic injury	Hypothesis-level only
Nucleostemin/nucleophosmin redistribution	Maintains nucleolar integrity and ribosome assembly	Disrupted during myocardial stress and associated with impaired cellular recovery	May provide sensitive early indicator of stress response	Not yet investigated in TBI

TBI, traumatic brain injury.

These features are presented as hypothesis-generating targets for future investigation in TBI-related cardiac dysfunction.

## 12 Conclusion

TBI is increasingly recognized as a systemic inflammatory condition with effects that extend beyond the CNS to include meaningful cardiac dysfunction. Clinical and experimental studies consistently show that TBI is associated with a range of cardiovascular abnormalities, from arrhythmias and biomarker elevations to changes in cardiac performance. Together, these findings support an association between TBI and clinically meaningful cardiac abnormalities that may extend beyond effects attributable solely to critical illness.

A recurring theme throughout this review is the variability in how these cardiac effects present. In many cases, abnormalities are subtle or clinically silent yet still carry important implications for recovery and long-term outcomes. This variability likely reflects the complex interplay between autonomic regulation, inflammatory signaling, and neuroendocrine responses following injury. Rather than acting independently, these processes may interact within a broader brain-heart framework that could influence cardiovascular responses following TBI.

Within this context, cerebrovascular integrity emerges as an important factor that may influence individual susceptibility to neurocardiac dysfunction. Alterations in CVR could affect the stability of the CAN, potentially amplifying downstream cardiovascular effects. Considering these vascular contributions alongside neural and cardiac mechanisms may provide a more comprehensive framework for understanding variability in clinical outcomes.

Overall, recognition of the brain-heart axis as an integrated biological system may provide a framework for understanding cardiac dysfunction after TBI. Continued integration of autonomic, vascular, and myocardial perspectives will be essential for identifying therapeutic targets and improving long-term cardiovascular outcomes.
